# Systemic diseases and their association with open-angle glaucoma in the population of Stockholm

**DOI:** 10.1007/s10792-021-02137-w

**Published:** 2021-11-29

**Authors:** Per Wändell, Axel C. Carlsson, Gunnar Ljunggren

**Affiliations:** 1 Department of Neurobiology, Care Sciences and Society, Division of Family Medicine and Primary Care, Karolinska Institutet, Alfred Nobels Allé 23, 141 83 Huddinge, Sweden; 2 Academic Primary Health Care Centre, Region Stockholm Region, Stockholm, Sweden

**Keywords:** Open-angle glaucoma, Administrative databases, Sex differences, Age differences, Epidemiology, Socio-economic areas

## Abstract

**Objective:**

We aimed to study open-angle glaucoma in association with somatic comorbidities in the total population of adults in Region Stockholm.

**Methods:**

The study population included all living persons aged 19 years and above who resided in Stockholm County, Sweden, on 1 January 2017 (*N* = 1 703 675). Subjects with specified diseases were identified with data from all registered consultations and hospital stays during 2008–2019. As outcome, the risk of being associated with a diagnosis of open-angle glaucoma was identified during 2012–2018. Analyses were performed by gender, controlling for age and socio-economic status. Age-adjusted odds ratios (ORs) with 95% confidence intervals (95% CI) for women and men with open-angle glaucoma, using individuals without this as referents, were calculated. Socio-economic status was assessed based on the neighbourhood the subjects lived in.

**Results:**

In total, 16,299 cases of open-angle glaucoma were identified during 2012–2018, 9204 women and 7095 men. Higher fully adjusted OR (95% CI) for risk of being associated with open-angle glaucoma was for women and men with diabetes 1.138 (1.074–1.207) and 1.216 (1.148–1.289), cancer 1.175 (1.120–1.233) and 1.106 (1.048–1.166), hypertension 1.372 (1.306–1.440) and 1.243 (1.179–1.311); and for women with thyroid diseases 1.086 (1.030–1.146), chronic lung diseases 1.153 (1.093–1.216), and inflammatory arthropathies 1.132 (1.006–1.275). Higher glaucoma incidence was observed in individuals residing in high socio-economic status neighbourhoods.

**Conclusion:**

The risk of glaucoma is increased in some somatic diseases, especially in individuals with diabetes, hypertension and cancer; and in higher socio-economic neighbourhoods as compared to lower socio-economic neighbourhoods.

**Supplementary Information:**

The online version contains supplementary material available at 10.1007/s10792-021-02137-w.

## Introduction

Glaucoma is one of the most important causes of low vision and blindness in the world. It was estimated that in 2020, 3.6 million people globally aged 50 years or above were blind due to glaucoma, with an age-standardized prevalence of 2.04 per 1000, and with 4.1 million people suffering from severe and moderate vision impairment, with an age-standardized prevalence of 2.29 per 1000 [1]. Actually, glaucoma was ranked as the second leading cause of blindness in the world, and the most common cause of irreversible blindness [1]. As the disease is mostly affecting older people, the contribution of glaucoma from moderate to severe visual impairment as well as to total blindness is higher in high-income regions with relatively older populations. Visual impairment including glaucoma is associated with both lower social participation [2], lowered quality of life [3, 4], and with increased mortality [5]. Besides, the burden of glaucoma in society is high and also increasing with the aging population [6].

The prevalence of glaucoma is estimated at 2% of the population above 40 years of age in Europe [7], but a Finnish study found a prevalence of 4.5% of the population aged 30 years and above [8]. Women have been shown to be more affected of visual impairment due to glaucoma [9, 10], with an estimated female-to-male ratio of 1.37 [11], suggesting that men and women should be analysed separately.

Primary open-angle glaucoma (POAG) is the most common type, accounting for three quarters of all glaucoma cases [12]. An earlier study from the north of Sweden found a prevalence of POAG among subjects aged 66 years to be 2.1% [13]. However, among patients with elevated intraocular pressure (IOP) only 9% became bilaterally visually impaired [14], thus indicating the importance of identifying patients early in the course of open-angle glaucoma to prevent visual impairment and blindness.

Even if patients with glaucoma are diagnosed, and attending their medical care by ophthalmologists, they frequently visit other care givers as well, as most patients with glaucoma are old. Thus, the comorbidity of patients with glaucoma is of importance to know for the ophthalmologist, as well as the presence of glaucoma among patients in other care forms [15]. In fact, POAG, with increased intraocular pressure [16], has been associated with hypertension [17–20], and diabetes [21–23]. Yet, lower systolic blood pressure, and lower perfusion pressure, are risk factors for POAG [24]. In a study from Taiwan, patients with POAG were compared with matched subjects regarding comorbidity, with the most important excess risks found for hypertension, hyperlipidemia, systemic lupus erythematosus, diabetes, hypothyroidism, as well as fluid and electrolyte disorders [25].

The aim of the present study was to study the association between open-angle glaucoma and some systemic somatic diseases in the total population of Region Stockholm. We also aimed to study the role of socio-economic status assessed by the neighbourhood status on the risk of open-angle glaucoma.

## Methods and study population

Region Stockholm today has over 2.2 million inhabitants, representing more than one-fifth of Sweden’s entire population. The region includes the capital city of Stockholm and several other cities and towns, as well as large rural areas and a sparsely populated archipelago. Region Stockholm Council is responsible for financing primary and secondary health care, mainly through taxes. Except a very small number of private clinics that operate without subsidies in Stockholm, all consultations and diagnoses are recorded and stored in a central database, the Stockholm Regional Health Care Data Warehouse (VAL). Besides consultations and diagnoses, VAL compiles and stores data on healthcare utilization from primary care, specialist open care, as well as in-hospital care [26]. As an indication of its accuracy and validity, VAL is used by the Region for updating the National Patient Register kept by the Swedish National Board of Health and Welfare (NBHW) as well as the annual benchmarking reports of the NBHW and the Swedish Association of Local Authorities and Regions [27]. Since 1997, diagnoses have been coded according to WHO’s International Classification of Diseases, 10th edition (ICD-10).

### Design

This is a cross-sectional study comparing people with a new diagnosis of open-angle glaucoma with people without this diagnosis with regard to the association with important systemic somatic diseases. Diagnoses were registered separately at discharge from hospital, or after a consultation in open care, and are thus clinically based. Only diagnoses from physicians were obtained.

### Study population

The present study population included all living persons 19 years of age and older who resided in Region Stockholm County on January 1, 2017 (*N* = 1 703 675). Data on all healthcare consultations in primary care, specialized open care, and in-hospital care between 2008 and 2019 were extracted from VAL. People with at least one visit or one hospital stay where a new diagnosis of open-angle glaucoma was registered during 2012–2018 were identified. Similarly, specified somatic diseases found in the Stockholm population from 2008 and onwards were identified.

### Outcome

We used the first recorded ICD code of H40.1 to identify a diagnosis of open-angle glaucoma.

### Studied systemic diseases

As background systemic somatic diagnoses (for ICD codes: see Supplementary files), we chose: thyroid diseases (hypo- and hyper-thyroidism), diabetes mellitus, cancer, hypertension, chronic heart diseases (including coronary heart disease, rheumatic and non-rheumatic valve disorders, cardiomyopathy, atrial fibrillation and congestive heart failure), stroke, chronic lower respiratory diseases (including asthma and COPD), gastro-intestinal diseases (including ulcers in stomach, duodenum or jejunum, and liver diseases) and inflammatory rheumatic disorders (including rheumatoid arthritis (RA) and SLE).

### Socio-economic status

Neighbourhood socio-economic status (NSES) was classified into three levels, i.e. high, middle or low, by the Mosaic tool. Mosaic is originally developed by a marketing company (Experian), in order to categorize consumers as making sale activities more effective. Mosaic could thus give a nuanced classification of socio-economic status, by using a multivariate modelling with over 400 variables to categorize population areas by postcodes. Data from 29 different countries are used and are useful also for classification of cohorts in epidemiologic research [28, 29].

### Ethics

All data we handled were anonymized, and none of the individuals could be identified. Management and analysis based on the VAL database is part of a continuous quality control of healthcare utilization in Region Stockholm, and ethical approval has been obtained from the regional ethical review board in Stockholm to study comorbidities with these data.

### Statistical methods

Standard descriptive statistics such as numbers and percentages of the total population (N) were used. Logistic regression was used in to calculate the odds ratios (OR) with 95% confidence intervals if the people with open-angle glaucoma had relatively more or fewer cases of the systemic diseases than the rest of the population, controlling for age and presented for each sex separately. Statistical analysis and data management were performed using SAS software, version 9.4 (SAS Institute Inc., Cary, NC).

#### Results

The demography of Region Stockholm with the number of men and women aged 19 years and older on 1 January 2017 is shown in Table 1, as well the neighbourhood socio-economic status by Mosaic. The number of women and men with open-angle glaucoma 2016 and 2012–2018 is shown in Table 2, with the incidence for 2016 and cumulative incidence 2012–2018 expressed as rate per 100,000. More women than men were diagnosed with glaucoma during the time periods, with an overall female-to-male ratio for 2016 of 1.16, and for 2012–2018 of 1.26. The number and percentages of subjects in different age groups with different diseases, divided according to the presence or absence of glaucoma, are presented in Table 3, showing a higher prevalence for all disease groups for glaucoma patients.

**Table 1:** Population (aged 19 years and older) in Region Stockholm 1 January 2017, by age-groups and sex, and by age groups and socio-economic level by Mosaic

Age group	Women	Men	All	High level	Middle level	Low level	Missing data of Mosaic level
	*N*(%)	*N*(%)	*N*(%)	*N*(%)	*N*(%)	*N*(%)	*N*(%)
19–49 yrs	502,683	514,898	1,017,581	454,807	195,098	350,151	17,525
	58.23	61.27	59.73	44.69	19.17	34.41	1.72
50–64 yrs	184,395	184,922	369,317	169,004	63,810	132,169	4334
	21.36	22.01	21.68	45.76	17.28	35.79	1.17
65–74 yrs	102,885	92,505	195,390	88,464	33,934	71,712	1280
	11.92	11.01	11.47	45.28	17.37	36.70	0.66
75–84 yrs	51,049	37,961	89,010	36,673	15,956	36,041	340
	5.91	4.52	5.22	41.20	17.93	0.49	0.38
85-w yrs	22,306	10,071	32,377	13,436	6760	12,072	109
	2.58	1.20	1.90	41.50	20.88	37.29	0.34
Total	863,318	840,357	1,703,675	762,384	315,558	602,145	23,588

Percentages by gender are shown vertically, and by Mosaic level shown horizontally (with missing data for Mosaic level)

**Table 2:** All patients with newly diagnosed open-angle glaucoma during 2016, and all newly diagnosed patients with open-angle glaucoma 2012–2018, by sex and age groups, with number of incident cases and incidence for 2016 and cumulated incidence for 2012–2012, expressed as rate per 100,000

Age group	Women 2016	Men 2016	All 2016	Women 2012–2018	Men 2012–2018	All 2012–2018
	*n* (rate)	*n* (rate)	*n* (rate)	*n* (rate)	*n* (rate)	*n* (rate)
19–49 y	24 (5)	47 (9)	71 (7)	198 (39)	312 (61)	510 (50)
50–64 y	161 (87)	176 (95)	337 (91)	1114 (604)	1219 (659)	2333 (632)
65–74 y	361 (351)	327 (353)	688 (352)	2835 (2756)	2430 (2627)	5265 (2695)
75–84 y	318 (623)	230 (606)	548 (616)	3029 (5934)	2115 (5572)	5144 (5779)
85 y-	154 (690)	78 (775)	232 (717)	2028 (9092)	1019 (10,118)	3047 (9411)
Total	1018 (118)	858 (102)	1876 (110)	9204 (1066)	7095 (844)	16,299 (957)

**Table 3:** Systemic somatic comorbidity groups in the whole Stockholm population, in all women and men as well as in men and women without and with newly diagnosed open-angle glaucoma in 2012–2018

Comorbid disorders	All women	Women without glaucoma	Women with glaucoma	All men	Men without glaucoma	Men with glaucoma
	*n*(%)	*n*(%)	*n*(%)	*n*(%)	*n*(%)	*n*(%)
Thyroid diseases	110,172	108,393	1779	19,886	19,512	374
		10.99	19.33		2.00	5.27
Diabetes mellitus	58,557	57,110	1447	78,873	77,273	1600
		5.79	15.72		7.94	22.55
Cancer	94,431	91,908	2523	87,782	85,407	2375
		9.32	27.41		8.78	33.47
Hypertension	221,338	215,179	6159	211,581	207,121	4460
		21.82	66.92		21.28	62.86
Heart diseases	82,618	79,525	3093	104,205	101,481	2724
		8.06	33.60		10.43	38.39
Stroke	32,967	31,745	1222	35,274	34,273	1001
		3.22	13.28		3.52	14.11
Lung diseases	122,971	121,197	1774	83,257	82,252	1005
		12.29	19.27		8.45	14.16
Gastrointestinal diseases	20,911	20,474	437	21,912	21,589	323
		2.08	4.75		2.22	4.55
Arthropathies, systemic tissue disorders	14,599	14,298	301	5183	5068	115
		1.45	3.27		0.52	1.62

The risk of open-angle glaucoma in different systematic diseases is shown in Table 4. In general, individuals with some somatic diseases showed a higher risk of open-angle glaucoma, i.e. in men and women with diabetes, hypertension and cancers, but also in women with thyroid diseases, chronic lung diseases, and inflammatory arthropathies or systemic tissue diseases. For heart diseases and stroke, the risk was lower in men and women, and for gastro-intestinal diseases also for men.

**Table 4:** Odds ratios (ORs), for the association between systemic somatic diseases and open-angle glaucoma during 2016, and during 2012–2018, with adjustment for age and neighbourhood socio-economic status in men and women

	Women 2016	Men 2016	Women 2012–2018	Men 2012–2018
	OR (95%CI)	OR (95%CI)	OR (95%CI)	OR (95%CI)
Thyroid diseases	1.111 (0.938–1.315)	1.171 (0.836–1.640)	**1.086 (1.030–1.146)**	1.029 (0.924–1.147)
Diabetes mellitus	**1.229 (1.023–1.475)**	1.160 (0.971–1.385)	**1.138 (1.074–1.207)**	**1.216 (1.148–1.289)**
Cancer	1.080 (0.906–1.288)	0.920 (0.766–1.103)	**1.175 (1.120–1.233)**	**1.106 (1.048–1.166)**
Hypertension	1.203 (1.048–1.380)	1.046 (0.904–1.208)	**1.372 (1.306–1.440)**	**1.243 (1.179–1.311)**
Heart diseases	**0.840 (0.712–0.989)**	**0.778 (0.658–0.920)**	**0.864 (0.823–0.908)**	**0.831 (0.787–0.878)**
Stroke	0.889 (0.688–1.148)	0.620 (0.458–0.838)	**0.908 (0.851–0.968)**	**0.845 (0.787–0.907)**
Lung diseases	**1.229 (1.031–1.465)**	1.002 (0.793–1.264)	**1.153 (1.093–1.216)**	1.011 (0.944–1.083)
Gastro-intestinal diseases	1.242 (0.833–1.851)	**0.461 (0.238–0.889)**	1.048 (0.949–1.157)	**0.828 (0.739–0.929)**
Arthropathies, systemic tissue disorders	1.221 (0.825–1.805)	1.328 (0.730–2.413)	**1.132 (1.006–1.275)**	1.166 (0.965–1.409)

Significant findings are shown in bold

The odds ratios of high and middle socio-economic levels vs. a low level are shown in Table 5. In general, the ORs were higher among high level areas among both women and men for all studied diseases.

**Table 5:** The role of (ORs) of neighbourhood socio-economic status (NSES) for the association between systemic somatic diseases and open-angle glaucoma during 2012–2018, by Mosaic, categorized into three groups: highest, middle, lowest), with lowest group as referent

	Women		Men	
	Highest vs lowest	Middle vs lowest	Highest vs lowest	Middle vs lowest
	OR (95%CI)	OR (95%CI)	OR (95%CI)	OR (95%CI)
Thyroid diseases	**1.077 (1.028–1.129)**	0.989 (0.932–1.050)	**1.083 (1.027–1.142)**	1.053 (0.983–1.127)
Diabetes mellitus	**1.086 (1.036–1.138)**	0.994 (0.936–1.055)	**1.095 (1.038–1.155)**	1.058 (0.988–1.133)
Cancer	**1.073 (1.024–1.125)**	0.988 (0.931–1.048)	**1.080 (1.024–1.139)**	1.051 (0.982–1.126)
Hypertension	**1.102 (1.051–1.155)**	1.001 (0.943–1.062)	**1.095 (1.038–1.154)**	1.059 (0.989–1.134)
Heart diseases	**1.072 (1.023–1.123)**	0.987 (0.930–1.048)	**1.078 (1.022–1.137)**	1.050 (0.981–1.125)
Stroke	**1.076 (1.027–1.128)**	0.989 (0.932–1.050)	**1.081 (1.026–1.140)**	1.052 (0.982–1.127)
Lung diseases	**1.082 (1.032–1.134)**	0.990 (0.933–1.051)	**1.083 (1.027–1.142)**	1.053 (0.983–1.127)
Gastro-intestinal diseases	**1.077 (1.028–1.129)**	0.989 (0.932–1.050)	**1.081 (1.025–1.140)**	1.052 (0.982–1.127)
Arthropathies, systemic tissue disorders	**1.077 (1.028–1.129)**	0.990 (0.932–1.050)	**1.083 (1.027–1.142)**	1.053 (0.983–1.128)

Significant findings are shown in bold

#### Discussion

The main finding of this study was that individuals with various somatic diagnoses, especially diabetes, hypertension and cancers, showed a higher risk of being diagnosed with open-angle glaucoma. The risk of open-angle glaucoma was higher in neighbourhoods with higher socio-economic status.

As regards disorders linked to the metabolic syndrome, we found a moderately increased risk among individuals with diabetes or hypertension, with ORs 1.1–1.4. This is in accordance with earlier studies, as the link between metabolic diseases and glaucoma is described [30], as both hypertension [17–20] and diabetes [21–23] are associated with POAG. One review concluded that “clinicians should consider adjusting the risk of glaucoma in patients who have chronic diabetes”, including dietary modifications, such as intake of increased fish oil [23]. Furthermore, in individuals with hypertension, a substantially lowered blood pressure may increase the risk of glaucoma complications with visual field loss [21]. In contrast, the risk of open-angle glaucoma in heart diseases and stroke showed a lower risk among men and women. The earlier study on comorbidities from the national study of Taiwan found a higher risk of heart disease and stroke in glaucoma patients with ORs of 1.2–1.5 [25]. However, the important link between heart diseases and stroke on the one hand, and open-angle glaucoma, might be hypertension. We have no good explanation for this discrepancy. Blood pressure might be lower in patients with an earlier heart disease or stroke, or the hypertension diagnosis might not be used in these patients, with other cardiovascular diseases.

As regards thyroid disorders, we found a rather small excess risk among women for when combining hyper- and hypothyroidism. The risk of open-angle glaucoma in patients with hypothyroidism has been reported previously [31].

Regarding chronic lung diseases, the risk of open-angle glaucoma was increased in women. Both asthma and COPD are of importance in relation to glaucoma, as beta blocker drops may affect the lung function [32]. The earlier mentioned article on comorbidities in patients with glaucoma found a slightly increased risk of these diseases with ORs of around 1.3 [25].

The risk among patients with cancer was also increased, in line with the findings by Lin et al. on the risk of having solid tumours without metastases among glaucoma patients [25].

Additionally, the risk of glaucoma was slightly decreased in men with gastrointestinal diseases compared that of the reference group. Again, we were interested in the risk of open-angle glaucoma in patients with these diseases, which may explain the disparate findings to the previously published comorbidity study [25].

The risk was also increased in women with RA or SLE. The earlier comorbidity study found a more pronounced risk of having especially SLE and to a lesser extent of RA among glaucoma patients [25].

There are some limitations with this study. We might have missed individuals without a recorded diagnosis. However, during the time period 94% of the inhabitants were registered with at least one visit. A Canadian study estimated the sensitivity of a glaucoma diagnosis to be 76% [33], and a UK study estimated the undetected cases of open-angle glaucoma to up to 67% [34]. However, overdiagnosis may also be at hand [35]. We used the ICD code H40.1 to identify a new diagnosis of open-angle glaucoma, in accordance with earlier studies. Out of all registered glaucoma diagnoses (ICD-10 codes H40-H42), open-angle glaucoma accounted for 30%, and “glaucoma suspect”, i.e. “ocular hypertension” (ICD code H40.0), 60%. Some studies have also included “ocular hypertension” (OHT) H40.0 [16], which we avoided. We chose to use logistic regression for both 2016 alone, as well as for the years 2012–2018. We chose this method with the intention to catch underlying diagnoses before the diagnosis of glaucoma. However, patients may have had the glaucoma disease for a considerable time before being diagnosed. There are also several strengths in the present study: the Swedish registers are known to be of high quality [36], and we have earlier used data from the VAL register in Region Stockholm with some examples cited [26, 37]. Furthermore, the study is truly population based, with data on nearly all healthcare visits of all residents of the Stockholm region. Furthermore, a glaucoma diagnosis could be expected to be set only by ophthalmologists.

In conclusion, we found several somatic diseases to be associated with a higher risk of open-angle glaucoma, especially diabetes, hypertension, and cancers. It is of importance to be aware of the increased risk of glaucoma in the care of these patients in order to provide them with timely therapy that enables them to see or to keep a good vision. Additionally, it would be of interest to study also psychiatric diagnoses in the same way as somatic diagnoses.

## Supplementary Information

Below is the link to the electronic supplementary material.

Supplementary file1 (DOCX 14 KB)
