# Supplementary material for: Systemic diseases and their association with open-angle glaucoma in the population of Stockholm
Source: Int Ophthalmol. 2021 Nov 29;42(5):1481–9. doi: 10.1007/s10792-021-02137-w (PMC9122867; doi:10.1007/s10792-021-02137-w)
Supplement: Supplementary file 1 — Supplementary file1 (DOCX 14 KB) [file 10792_2021_2137_MOESM1_ESM.docx]

**Supplementary material**

Co-morbid conditions

We used the following co-morbidities (ICD-code): Thyroid disorders (including hypothyroidism E03 and hyperthyroidism E05); Diabetes mellitus (E10-E13, E14); Malignant neoplasms (C00-C46, C50-C58, C60-C72, C76-C96); Hypertension (I10-I13 and I15); Chronic heart diseases (chronic rheumatic heart diseases I05-I08, coronary heart disease I20-I25, non-rheumatic valve disorders I34-I38, cardiomyopathy I42, atrial fibrillation I48 and congestive heart failure I50); Stroke (I60-I69); Chronic lower respiratory diseases (J44-J46, including asthma and COPD); Gastro-intestinal diseases (including gastro-jejunal ulcers K25-K28, and diseases in liver K70-K77); and Arthropathies (including rheumatoid arthritis M05-M06 and systemic lupus erythematosus M32).
